# Detection of serum HER2 in patients treated with neratinib or trastuzumab: analysis of the I-SPY Trial

**DOI:** 10.3389/fonc.2025.1605120

**Published:** 2025-07-24

**Authors:** Mark Hensley, Justin Lengfeld, Steven Stoesz, Michelle Edwards, Franklin Pass, Gillian L. Hirst, Lamorna Brown-Swigart, Laura van ‘t Veer, Laura J. Esserman, Heather Beckwith, Douglas Yee

**Affiliations:** ^1^ Hensley Biostats, Seattle, WA, United States; ^2^ Martell Diagnostic Laboratories, Roseville, MN, United States; ^3^ Department of Surgery, University of California, San Francisco, San Francisco, CA, United States; ^4^ Department of Medicine, Division of Hematology, Oncology, and Transplantation, University of Minnesota, Minneapolis, MN, United States; ^5^ Masonic Cancer Center, University of Minnesota, Minneapolis, MN, United States

**Keywords:** HER2, biomarkers, neoadjuvant therapy, neratinib, trastuzumab

## Abstract

**Purpose:**

Drugs targeting human epidermal growth factor receptor 2 (HER2) have fundamentally changed the way breast cancer is treated. Measurement of HER2 expression has become increasingly important with the approval of therapies targeting a HER2-low population. Furthermore, predictive biomarkers for HER2 response would aid the clinical use of these drugs, and a blood-based assay of HER2 could provide important information for therapeutic options for patients.

**Methods:**

To evaluate serum HER2 (sHER2) as a potential biomarker for breast cancer response, we examined the serum samples from patients treated with neratinib or trastuzumab combined with paclitaxel obtained from the I-SPY2 neoadjuvant trial. This trial included both HER2-positive and HER2-negative/low tumors.

**Results:**

Of the patients with HER2-negative tumors, 26% had elevated sHER2, while 56% of the HER2-positive patients had elevated sHER2. The sHER2 levels declined with neoadjuvant therapy, and most patients had a clinical response to therapy. However, the sHER2 decline was not predictive of pathologic complete response.

**Conclusion:**

sHER2 was detected in patients with HER2 tissue-positive and tissue-negative tumors. Further study will be needed to determine whether sHER2 is associated with patients with tumors that are HER2-low or ultralow and whether changes in sHER2 over time could predict response to HER2-targeted drugs.

**Clinical Trial Registration:**

clinicaltrails.gov, identifier NCT01042379.

## Introduction

Targeting of human epidermal growth factor receptor 2 (HER2) is one of the seminal advances in breast cancer therapy. Breast cancers with high expression and gene amplification of HER2 are vulnerable to multiple drugs, and targeting HER2 expression remains an active area for new drug development (1).

HER2 is a 185-kDa transmembrane receptor with extracellular (ECD), transmembrane, and tyrosine kinase domains. It has long been known that proteases can cleave the receptor to free the 95-kDa ECD that is detectable in a patient’s serum. While there was initial concern that the ECD of HER2 (serum human epidermal growth factor receptor 2, sHER2) could affect the pharmacokinetic profile of trastuzumab, this was not shown in clinical studies. Nor was it evident that sHER2 is a predictive biomarker for the response to HER2 targeting. Thus, sHER2 detection has not been used in the identification of patients eligible for HER2 targeting strategies or in the monitoring of patients with HER2-positive tumors (6).

Recently, measurement of the tissue HER2 expression has gained increasing importance given the approval of new antibody–drug conjugates (ADCs) for patients with lower levels of HER2 than required for first-generation monoclonal antibodies such as trastuzumab (3, 7). Thus, HER2-low and ultralow tumors now have new therapeutic options. The sHER2 levels may be correlated with the tissue HER2 expression and have clinical impacts. The relationship between sHER2 and the tissue expression of HER2 is not well characterized.

To further study the tissue expression of HER2 and the detection of sHER2, we examined patients enrolled on the I-SPY2 clinical trial (4). This neoadjuvant trial evaluates novel agents in combination with paclitaxel in patients with high-risk tumors, with the primary endpoint being pathologic complete response (pCR). It uses a Bayesian randomization scheme to optimize responses. We examined patients who were treated with neratinib and paclitaxel, as this arm was open to all patients eligible for I-SPY2. While neratinib is approved for the treatment of HER2-positive breast cancer, it also has activity against the epidermal growth factor receptor (EGFR) tyrosine kinase (10). Because EGFR may have a biological role in non-HER2-positive breast cancer, patients without HER2 amplification or overexpression were enrolled in this study. Using this dataset provided an opportunity to examine sHER2 in patients with HER2-negative tumors.

As reported, neratinib and paclitaxel followed by doxorubicin and cyclophosphamide (AC) was a successful combination only in the HER2-positive subgroup (8). In the HER2-negative subgroup, there was no evidence of benefit of the addition of neratinib to paclitaxel compared with paclitaxel alone. Further analysis confirmed that HER2 signaling is associated with the best responses to HER2-targeted therapy (5). In our analysis, patients with HER2-positive tumors received trastuzumab and paclitaxel (control group), and these patients were also studied. This study evaluates the expression of sHER2 in comparison to tissue levels and examines any potential role as a predictive biomarker in response to this therapy.

## Methods

### Patients

The results for the patients treated with neratinib in the I-SPY Trial have been previously reported (8). The tissue expression of HER2 was determined locally, and all patients with HER2-positive tumors were eligible for the study. HER2-negative patients were required to have a high-risk result in the 70-gene assay (MammaPrint^®^). In this study, patients with HER2-positive tumors received 12 weeks of either neratinib and weekly paclitaxel or trastuzumab and paclitaxel. Patients with HER2-negative tumors received neratinib with paclitaxel or paclitaxel alone. After 12 weeks of this randomized therapy, all patients received AC for an additional four cycles, either 8 or 12 weeks depending on whether AC was given in a dose-dense fashion. Serum was obtained at four time points: T0 is prior to treatment, T1 is 3 weeks after starting therapy, T2 is at 12 weeks after completion of the paclitaxel therapy, and T3 is after completion of AC. For this analysis, the HER2-negative patients who only received paclitaxel were not examined.

### ELISA

The HERTEST serum HER2 immunoassay is a solid-phase sandwich ELISA designed to measure human HER2 protein in serum samples. The immunoassay utilizes a rabbit monoclonal antibody for capture and a different biotinylated rabbit monoclonal antibody for detection. Both the capture and detector reagents specifically bind to different regions of the ECD of HER2 protein. The capture antibody was immobilized on the interior surface of microtiter plate wells. After washing away any unbound proteins, the immobilized HER2 was then detected with the biotinylated detection antibody. The amount of detector antibody bound was measured with a streptavidin/horseradish peroxidase conjugate. Following a wash, a substrate solution was added and the color development measured using a microplate spectrophotometer. An elevated sHER2 assay was >13.3 ng/ml. Normal range was determined by studying 120 normal sera as detailed in the patent application for the assay (US-20240210403-A1).

For each patient, pCR and the residual cancer burden (RCB) (9, 11) were determined.

### Statistical analysis

The odds ratios of the measures of sHER2 at various time points *vs*. the clinical outcomes were calculated using logistic regression. Correlations of the measures of sHER2 at various time points *vs*. the clinical outcomes were determined using Pearson’s correlation coefficients. The beta coefficients of the measures of sHER2 at various time points *vs*. the clinical outcomes were determined by linear regression. Significance testing for differences in the levels of sHER2 by estrogen receptor (ER) status was determined with a *t*-test. Tests for group differences in the Kaplan–Meier curves of the clinical outcomes over time were conducted using Cox proportional hazard models. All analyses were performed using Stata v17.

## Results

### Detection of sHER2

A total of 130 patients who had a baseline and at least one other available serum sample were studied. **Table 1** demonstrates the association between elevated sHER2 and tissue HER2 expression. Of the 130 patients, 50 were tissue HER2-negative and 13 (26%) had elevated sHER2. When examined by ER status, most of the sHER2-positive patients had ER-negative tumors (10/13).

**Table 1:** Serum human epidermal growth factor receptor 2 (sHER2) detection by tissue HER2 and estrogen receptor (ER) status.

Tissue HER2-negative	50
sHER2 positive	13 (26%)
ER Negative	10
ER Positive	3
sHER2 negative	37 (74%)
ER Negative	22
ER Positive	15

Tissue HER2-Positive	80
sHER2 positive	45 (56%)
ER Negative	15
ER Positive	30
sHER2 negative	35 (44%)
ER Negative	14
ER Positive	21
**Total**	**130**

*The doses of pyrazinamide and ethambutol were increased in accordance with weight-based dosing recommendations

Of the 80 patients who tissue HER2-positive, 56% also had elevated sHER2. In these patients, approximately half of the sHER2 patients had ER-negative tumors (15/29). In this group of sHER2-positive subjects, more than half of those ER-positive (30/51) also had elevated sHER2. Thus, the assay detected sHER2 in patients with tissue-positive and tissue-negative HER2 tumors.

### Change in sHER2 over the course of therapy

**Figure 1:**
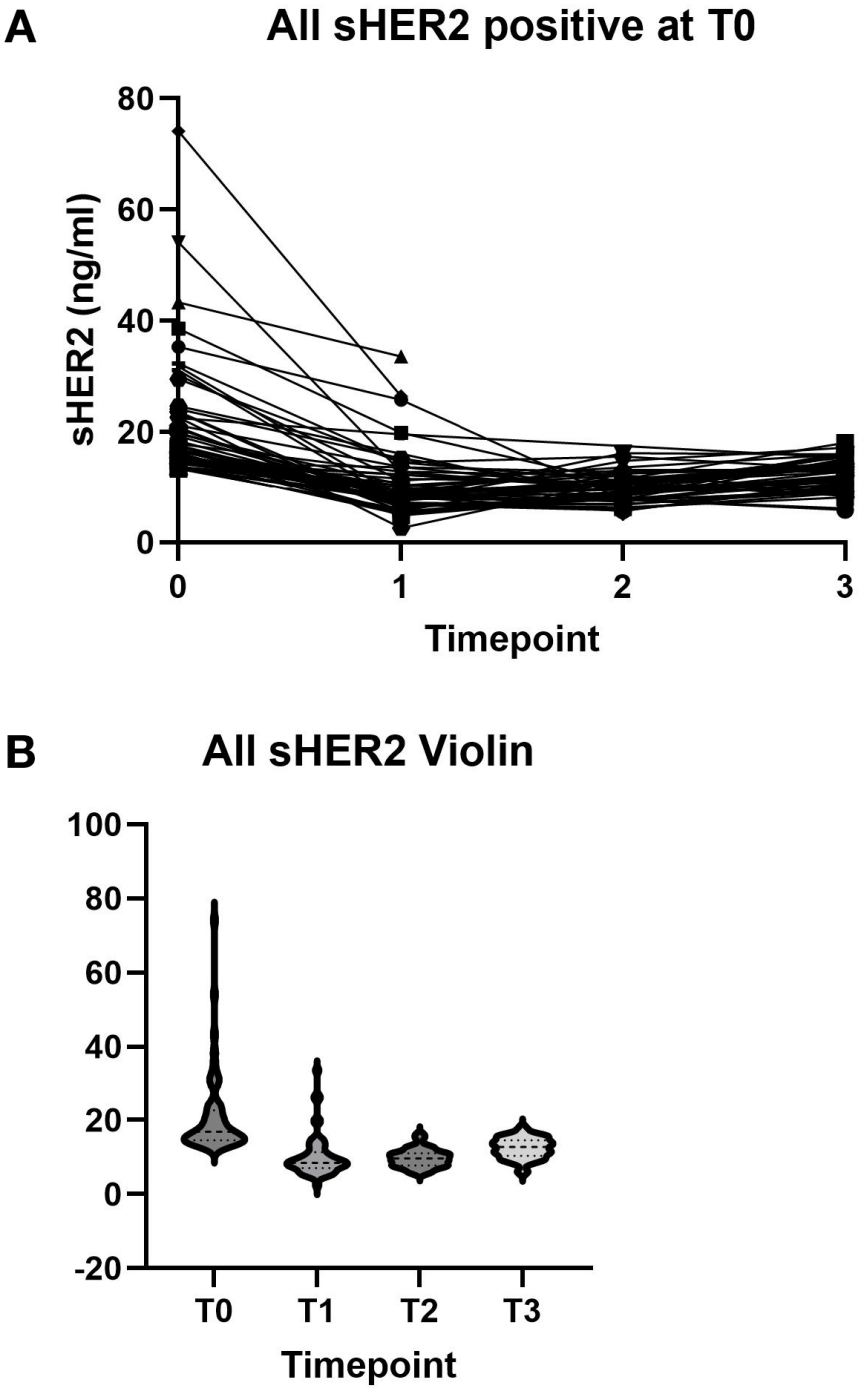
**(A)** Serum human epidermal growth factor receptor 2 (sHER2) levels at baseline and after treatment for individual patients. **(B)** sHER2 distribution at each time point.

**Figure 1A** shows the results of sHER2 with positive tumors at T0. As shown, most of the sHER2 values decreased after the initiation of therapy. **Figure 1B** demonstrates the distribution of sHER2 at each time point. In these treatment arms, 1% of the control group did not complete therapy due to adverse events, while 11% of the neratinib group discontinued treatment. There were no discontinuations for disease progression in the control group, while 6 of the 115 (5%) in the neratinib group discontinued therapy for progression (8). Of these patients with progression, five had available serum samples, and all of these patients had baseline levels in the normal range.

We next examined whether the changes in sHER2 correlated with the response to therapy. I-SPY2 examined RCB and demonstrated that favorable long-term outcomes were observed for patients with pCR (12). For patients with HER2-positive tumors, a small amount of RCB, characterized as RCB-1, also confers a favorable long-term outcome (11).

**Figure 2:**
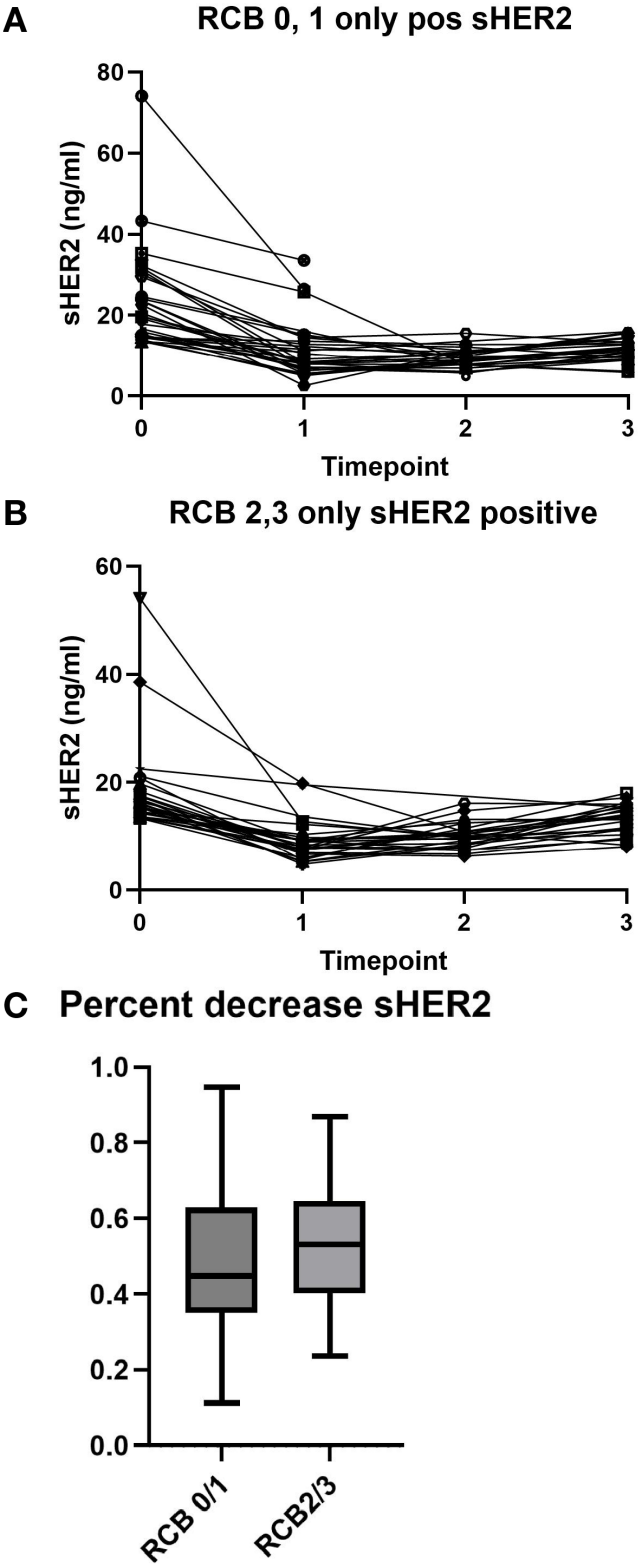
Serum human epidermal growth factor receptor 2 (sHER2) levels by neoadjuvant treatment response. **(A)** sHER2 changes in patients with a favorable response [residual cancer burden (RCB) 0 or 1]. **(B)** sHER2 levels in patients with an unfavorable response (RCB 2 or 3). **(C)** Percentage decrease in the sHER levels by RCB class. The median is represented by the *horizontal line*, the *box* outlines the 25th–75th percentile, and the *whiskers* show the minimum and maximum values.

Thus, we examined the sHER2 results in two categories: RCB-0/1 and RCB-2/3. **Figure 2A** shows that patients with a good response (RCB-0/1) had a decline in sHER2. However, patients with a lesser response (RCB-2/3) also had a decreased sHER2 (**Figure 2B**). For those patients who achieved RCB-0/1, 21/32 (65.6%) had a decline in sHER2 to the normal range during any treatment time point. For those with RCB-2/3, fewer (13/27, 48%) patients achieved and maintained a normal level of sHER2 after treatment.

To determine whether a percentage change in sHER2 is associated with response, this was examined for the two response groups. As shown in **Figure 2C**, there was no clear association between a percentage decrease in sHER2 and treatment response in the pCR group.

## Discussion

We examined sHER2 in the serum of patients who received neoadjuvant chemotherapy in the I-SPY2 clinical trial testing neratinib in addition to paclitaxel for both tissue HER2-positive tumors and negative tumors.

We found that a significant number of both tissue HER2-positive and HER2-negative patients had elevated sHER2 levels. While patients with tissue HER2-positive tumors had a higher rate of sHER2 (56%), it is still notable that 26% of the tissue HER2-negative patients had elevated sHER2. Other investigators have demonstrated sHER2 detection in patients with HER2-low tumors (2). Given the expanding indication for HER2-targeted ADCs in these “HER2-low” tumors, further study is needed to determine whether sHER2 can be used to identify patients eligible for treatment with these newer drugs.

We also found that sHER2 decreased after therapy. Numerically, more patients who achieved pCR or RCB-1 obtained and maintained normal levels compared with patients who had poorer responses, although this did not reach statistical significance. A larger cohort will be necessary to determine whether obtaining normal sHER2 levels predicts better responses. We did not find a relationship between the percentage change in sHER2 and response, suggesting that the normalization of sHER2 may be a more relevant predictive biomarker of response.

The strengths of this study include the ability to follow sHER2 over time, robust measures of clinical response, and the study of effective HER2 therapies. Its limitations include the relatively small sample size and a treatment regimen that is currently not the standard of care for HER2-positive patients. Only HER2-positive patients [by immunohistochemistry (IHC) or *in situ* hybridization (ISH)] were included in this analysis. The IHC values for HER2 were obtained locally and were not available for analysis. This study did not measure the sHER2 changes in the HER2-negative paclitaxel control arm to determine whether a decline in sHER2 in the HER2-negative subgroup could also be detected after treatment with paclitaxel alone.

In conclusion, it was found that sHER2 can be detected in patients who have HER2-positive and HER2-negative tumors. There was a trend correlating the sHER2 levels (achievement of a normal value) with favorable outcomes, although larger sample sizes will be necessary to demonstrate this. The measurement of sHER2 during the course of treatment could provide a biomarker associated with response to HER2-targeted therapies. sHER2 can also be detected in patients with HER2-negative (non-amplified and IHC-negative) tumors. The measurement of sHER2 in patients with HER2-low and ultralow tumors might help identify those who could benefit from the newer HER2-targeted therapies effective for these tumors.

## Data Availability

The datasets presented in this study can be found in online repositories. The names of the repository/repositories and accession number(s) can be found below: https://www.cell.com/cancer-cell/fulltext/S1535-6108(22)00216-1#secsectitle0075.
